# ﻿New fungal genus, three novel species and one new record from mangroves, with reclassification of *Melanconiella* (Melanconiellaceae) species

**DOI:** 10.3897/mycokeys.116.137351

**Published:** 2025-04-07

**Authors:** Carlo Chris S. Apurillo, Chayanard Phukhamsakda, Kevin D. Hyde, Vinodhini Thiyagaraja, E. B. Gareth Jones

**Affiliations:** 1 Key Laboratory of Phytochemistry and Natural Medicines, Kunming Institute of Botany, Chinese Academy of Sciences, Kunming, Yunnan 650201, China; 2 School of Science, Mae Fah Luang University, Chiang Rai 57100, Thailand; 3 Center of Excellence in Fungal Research, Mae Fah Luang University, Chiang Rai 57100, Thailand; 4 Department of Science and Technology-Center for Research in Science and Technology (CReST), Philippine Science High School-Eastern Visayas Campus, Palo, Leyte 6501 Philippines; 5 Department of Botany and Microbiology, College of Science, King Saud University, P.O. Box 2455, Riyadh, 11451, Saudi Arabia; 6 Nantgaredig, 33B St Edwards Road, Southsea, Hants., UK PO5 3DH, UK

**Keywords:** Fungi, mangrove, novel species, Pranburi, *Pseudomelanconiella*, saprobic fungi, taxonomy

## Abstract

Mangrove ecosystems, located in the land-sea interface, host a diverse array of fungi. In this paper, we introduce a novel genus, three novel species and one new record of fungi collected from mangrove environments in Pranburi, Prachuap Khiri Khan, Thailand. We establish *Pseudomelanconiella* as a new genus in Melanconiellaceae, to accommodate *Pseudomelanconiellamangrovei*, a saprobe from submerged decomposing wood of *Avicenniamarina*. Phylogenetic analysis indicates its close relation with *Septomelanconiella*, but they differ in the morphology of the conidia. Additionally, our analysis of Melanconiellaceae led to the reclassification of *Melanconiellaloropetali* to *Sinodisculaloropetali* and synonymizing *Sinodisculacamellicola* and *Melanconiellacamelliae*. This paper also introduces two other novel species: *Peroneutypahibisci*, a saprobe found on *Hibiscustiliaceus* and *Pseudochaetosphaeronemabruguierae* from *Bruguieracylindrica*, the first species in this genus reported as a mangrove fungus. A new record of *Rimoramangrovei* from *Ceriopstagal* is also reported. These discoveries emphasize the rich fungal diversity in mangrove ecosystems supporting further exploration of this unique environment.

## ﻿Introduction

Mangroves, found in unique intertidal habitats, are vital ecosystems hosting diverse flora and fauna. Though they cover only 1% of tropical forests, they support various plants and animals including mammals, birds, fish, and insects (Jia et al. 2020). Mangroves are also diverse hosts to a high number of fungal species (Norphanphoun et al. 2018; Devadatha et al. 2021).

The fungal kingdom, with an estimated 2.2–13 million species has a rich evolutionary history deeply intertwined with that of terrestrial, aquatic, and marine ecosystems (Hyde and Jones 1988; Wu et al. 2019; Hyde et al. 2020, 2024; Phukhamsakda et al. 2022). However, only about 10 percent of these have been described and reported (Hyde et al. 2024). This is the lowest percentage of described species among the eukaryotes compared to plants (83–96%) and animals (19%) (Wang et al. 2020; Bhunjun et al. 2022; Phukhamsakda et al. 2022). This highlights the need to continuously survey for fungal species, especially in unique ecological niches such as the mangrove environment.

Fungal study in mangroves began in the 1920s with the report of Stevens (1920) from mangroves in Puerto Rico, followed by the work of Cribb and Cribb (1955) in Australia. Kohlmeyer (1968) listed 75 species of mangrove fungi, noting their taxonomic diversity and host preferences. Later, Hyde and Jones (1988) documented 90 species of intertidal mangrove fungi across 26 tree species. Many species are widespread across different ocean basins, indicating their adaptability to diverse environmental conditions (Hyde and Jones 1988). Jones and Alias (1997) identified 268 mangrove fungi species, observing limited host-specificity but some host preferences, indicating a nuanced relationship between fungal communities and host plant diversity within the mangrove ecosystem. They suggested higher fungal diversity in Asian tropics due to greater host diversity, although this assertion is somewhat complicated since more studies were being conducted in Asia (Hyde and Lee 1995; Jones and Alias 1997).

Schmit and Shearer (2003) published the first checklist of mangrove fungi recognizing 625 species, including some freshwater and terrestrial taxa. Devadatha et al. (2021) updated this to 850 species, marking an increase of more than 200 taxa within the 18-year between the checklists. However, Devadatha et al. (2021) focused exclusively on marine fungi from mangrove substrates, excluding those from freshwater and terrestrial environments. Over the years, the number of fungi associated with mangroves has steadily increased due to a wider interest in their study, especially in bioprospecting for bioactive compounds useful in medicine, agriculture, and biotechnology (Tan et al. 2015; Dela Cruz et al. 2020; Cadamuro et al. 2021; Sopalun et al. 2021; Chen et al. 2022).

In this study, we introduce one new genus, three novel species and one new record of fungi from mangroves in Prachuap Khiri Khan province in Thailand.

## ﻿Methods

### ﻿Collection, observation, and isolation

Mangrove samples were collected from Pranburi Forest Park (No. 0907.4/23579) and the Pranburi River area in Prachuap Khiri Khan, Thailand. The samples included dead branches attached to mangroves and decomposing branches submerged in brackish water. They were sealed in plastic bags and transported to the laboratory at the Center of Excellence in Fungal Research, Mae Fah Luang University in Chiang Rai, Thailand.

Fungi on the samples were examined using a stereomicroscope, and their morphological features were documented with a Nikon Eclipse Ni compound microscope equipped with a Nikon DS-Ri2 camera (Nikon, Japan). Measurements were taken using Tarosoft® Image Framework software calibrated for the microscope. Photo plates were created using Adobe Photoshop 24.0 (Adobe Systems, USA).

To cultivate the fungi, single-spore isolation technique was employed (Chomnunti et al. 2014; Senanayake et al. 2020). Cultures were incubated at room temperature for 2–4 weeks and then used for DNA extraction. These cultures were deposited in the Mae Fah Luang University Culture Collection (MFLUCC). Additionally, herbarium samples were deposited at the Mae Fah Luang University Herbarium (MFLU). Novel fungal species were registered in Index Fungorum (2024) and Faces of Fungi (Jayasiri et al. 2015).

### ﻿DNA extraction and PCR

DNA extraction from mycelia of pure fungal cultures was carried out using the E.Z.N.A® Tissue DNA kit (Omega Biotek, USA) according to the manufacturer’s instructions. Initial tissue lysis was performed using the Qiagen Tissuelyzer (Qiagen, Netherlands), with the samples mixed with lysis solution. Subsequent steps followed the protocol outlined in the extraction kit.

Multi-locus amplification was conducted on all isolates. PCR reactions were prepared in 25 µl volumes, comprising 21 µl of Vazyme® Rapid Taq Master Mix, 1 µl each of forward and reverse primer, and 2 µl of template DNA. The internal transcribed spacer region (ITS) and different loci were amplified for the isolates using distinct primers (Table 1). For *Pseudomelanconiellamangrovei*, nuclear large subunit ribosomal DNA (LSU), RNA polymerase II second largest subunit (*rpb2*), and translation elongation factor 1-alpha (*tef1-α*) were amplified with *tef1-α* using EF1-728F/TEF1-LLeReV primers. For *Peroneutypahibisci*, beta-tubulin (*tub2*) was also amplified. For *Pseudochaetosphaeronemabruguierae* and *Rimoramangrovei*, LSU, 18s small subunit ribosomal gene (SSU), and *tef1-α* were amplified, using EF1-983F and EF1-2218R primers for *tef1-α*. For *R.mangrovei*, only LSU, SSU and TEF were used for the phylogenetic analysis, as detailed in the results section. The list of primers and PCR conditions for each primer pair is provided in Table 1.

**Table 1.** Primers and PCR conditions used in the study.

Locus	Primers	Sequence (5’-3’)	Reference	PCR Conditions
ITS	ITS5	GGAAGTAAAAGTCGTAACAAGG	White et al. (1989)	95 °C, 5 min; 35 cycles of 95 °C 45 s, 53 °C 45 s, 72 °C 2 min; 72 °C 5 min
	ITS4	TCCTCCGCTTATTGATATGC		
LSU	LR0R	ACCCGCTGAACTTAAGC	Vilgalys and Hester (1990)	94 °C 3 min;; 35 cycles of 94 °C 1 min, 52 °C 50 s, 72 °C 1 min; 72 °C 10 min
	LR5	ATCCTGAGGGAAACTTC		
*rpb2*	fRPB2-5F	GAYGAYMGWGATCAYTTYGG	Liu et al. (1999)	94 °C 3 min;; 35 cycles of 94 °C 1 min, 52 °C 50 s, 72 °C 1 min; 72 °C 10 min
	fRPB2-7CR	CCCATRGCTTGYTTRCCCAT		
SSU	NS1	GTAGTCATATGCTTGTCTC	White et al. (1989)	95 °C 2 min; 35 cycles of 95 °C 30 s, 55 °C 50 s, 72 °C 1 min; 72 °C 10 min
	NS4	CTTCCGTCAATTCCTTTAAG		
*tef1-α*	EF1-728F	CATCGAGAAGTTCGAGAAGG	Carbone and Kohn (1999)	95 °C 2 min; 35 cycles of 95 °C 30 s, 56 °C 45 s, 72 °C 1 min; 72 °C 8 min
	TEF1-LLeReV	AACTTGCAGGCAATGTGG	Jaklitsch et al. (2005)	
	EF1-983f	GCYCCYGGHCAYCGTGAYTTYAT	Rehner and Buckley (2005)	95 °C 2 min; 35 cycles of 95 °C 30s, 55 °C 50s, 72 °C 1 min; 72 °C 10 min
	EF1-2218R	CCRAACRGCRACRGTYYGTCTCAT		
*tub2*	Bt2a	GGTAACCAAATCGGTGCTGCTTTC	Glass and Donaldson (1995)	95 °C 5 min; 35 cycles of 94 °C 30 s, 54 °C 30 s, 72 °C 1 min; 72 °C 8 min
	Bt2b	ACCCTCAGTGTAGTGACCCTTGGC		

Following PCR, the resulting products were electrophoresed on a 1% agarose gel to verify the sizes of the amplicons. If single bands matching the expected sizes were observed in the gel, the PCR products were forwarded for Sanger sequencing at Sangon Biotech (China).

### ﻿Phylogenetic analysis

The sequences were assembled using Seqman II v. 5.0 to produce a contig. Subsequently, the contigs were searched in the Basic Local Alignment Search Tool (Madden 2003) to check for closely related sequences. Based on the results, related sequences to the isolates were retrieved based on previous studies. The National Center of Biotechnology Information (NCBI) Nucleotide database was checked to ensure the inclusion of all related species with molecular data in the analysis. The accession numbers of the sequences used in this study are shown in Suppl. material.

The sequences of the isolates and related species were aligned using Multiple Alignment using Fast Fourier Transform (MAFFT) version 7.4 with strategy set to auto, a gap extend penalty of 0.123, a gap opening penalty of 1.53, with adjust direction selected (Katoh and Standley 2013). The alignment was cleaned up by Block Mapping and Gathering with Entropy (BMGE; Criscuolo and Gribaldo 2010), with the following settings: DNAPAM matrix, a sliding window size of 3, gap rate cut-off of 0.5, maximum entropy threshold of 0.5, and minimum block size of 5. MAFFT and BMGE were performed on the NGPhylogeny site (Lemoine et al. 2019).

The aligned sequences were then subjected to maximum likelihood (ML), maximum parsimony (MP), and Bayesian inference (BI) analyses. For ML, RAxML-HPC2 on ACCESS v. 8.2.12 (Stamatakis 2014) was utilized, using the default GTRGAMMA model and 1,000 bootstrap iterations. For MP, PAUP on XSEDE v.4.168 (Swofford 2002) set to 1,000 bootstrap iterations was employed.

Model testing was conducted prior to Bayesian analysis using MEGA v. 11 (Tamura et al. 2021), and specific models were applied during the analysis. For the multi-locus analysis, specific models for each partition of the concatenated sequences were specified in the command block. Bayesian posterior probability analysis was conducted using Markov chain Monte Carlo (MCMC) sampling in MrBayes v.3.2 on XSEDE (Ronquist et al. 2012), with four simultaneous Markov chains run for 10,000,000 generations, sampling every 1,000^th^ generation. The run discarded 25% of the trees as the burn-in phase, using the remaining trees to compute the posterior probability (PP) in the consensus tree. Trace files of individual and combined runs were assessed using Tracer v.1.7.2 (Rambaut et al. 2018).

Phylogenetic analyses were initially performed on individual loci before multi-locus analyses were conducted. Trees generated from individual locus and multi-locus analyses for each taxon were compared, and only reported if they exhibited similar topologies. Trees were visualized using FigTree v.14.4 (Rambaut 2018), and then edited using Adobe Illustrator v.27.0 (Adobe Systems, USA), combining the bootstrap values from ML and MP with the posterior probabilities (PP) from Bayesian analysis. The decisions as to whether to introduce new genera or species follow Jeewon and Hyde (2016) and Maharachchikumbura et al. (2021).

## ﻿Results

### ﻿Sordariomycetes O.E. Erikss. & Winka

**Diaporthales Nannf.**

**Melanconiellaceae Senan., Maharachch. & K.D. Hyde**

Phylogenetic analysis for *Pseudomelanconiella* isolates was performed using ITS, LSU, *rpb2* and *tef1-α* loci. The ML, MP and Bayesian multi-locus analysis consisted of a total of 3,957 characters, including gaps in the concatenated sequence. The lengths of each region were as follows: ITS (1-592), LSU (593-1,469), *rpb2* (1,470-2,631), *tef*1-α (2,632-3,957).

**Figure 1.**
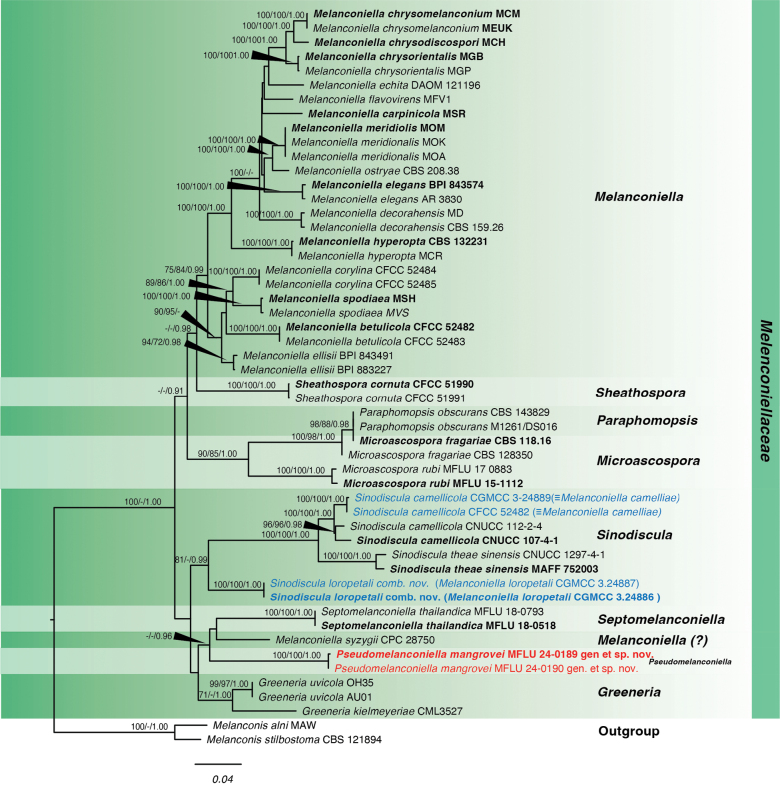
Phylogram of Melanconiellaceae based on a combined analysis of ITS, LSU, *rpb2*, and *tef1-α*. Values above the branches indicate bootstrap support from maximum likelihood (ML) and maximum parsimony (MP) equal to or above 70 and Bayesian posterior probabilities (PP) equal to or above 0.90. Novel species are in red, and species for synonymization and reclassification are in blue. Sequences from type species are indicated in bold. The tree is rooted with *Melanconisalni* MAW and *Melanconisstilbostoma* CBS 121894.

For ML analysis, the alignment had 1,527 distinct alignment patterns with 23.52% gaps and undetermined characters. The best-scoring tree (shown in Fig. 1) had a final optimization likelihood of -25,438.87, tree length of 1.744101 and alpha of 0.239245. The base frequencies are: A = 0.227229, C = 0.273128, G = 0.273187 and T = 0.226456, with the following substitution rates: AC = 1.270018, AG = 3.717788, AT = 1.313118, CT = 5.037061, and GT = 1.00000.

The MP analysis of the multi-locus data included 3,957 characters, with 2,470 constant characters, 1,318 parsimony-informative and 169 parsimony-uninformative characters. The most parsimonious tree had a similar topology to the maximum likelihood tree (Fig. 1), with the *Pseudomelanconiella* clade in the same position on the tree.

For the Bayesian analysis, different evolutionary models were used for each locus based on the results from MEGA: Kimura 2-parameter with gamma distribution and proportion of invariable sites (K2P+G+I) for ITS, General Time Reversible model with gamma distribution (GTR+G) for LSU and General Time Reversible model with gamma distribution and proportion of invariable sites (GTR+G+I) for *rpb2* and *tef1-α*. The final average standard deviation of split frequencies after the total MCMC generations is 0.002019. Effective sample size (ESS) values for all factors of the combined trace files ranged from 7,577 to 15,002 with the trace plots showing two independent runs have converged. The topology of the tree from the Bayesian analysis is similar to the one obtained from the maximum likelihood analysis.

Based on the ML, MP, and BI analyses, which included sequences from species belonging to Melanconiellaceae, *Pseudomelanconiella* isolates (MFLU 24-0189, MFLU 24-0190) formed a distinct clade closely related to *Septomelanconiella* (Fig. 1). Although the node that separates *Pseudomelanconiella* and *Septomelanconiella* has low support in ML and MP, it has a good support for BI and is consistently recovered in all trees generated in the three analyses. Furthermore, the base differences of ITS, LSU and *rpb2* between *Septomelanconiella* and *Pseudomelanconiella* are higher than the least base difference between genera in Melanconiellaceae. Morphologically, *Pseudomelanconiella* differs from *Septomelanconiella*, as detailed in the taxonomy notes. *Pseudomelanconiella* is also phylogenetically distant from *Sinodiscula*, the most recent genus to be added to Melanconiellaceae.

Our analysis shows that three *Melanconiella* species do not group within the *Melanconiella* clade. Thus, *Melanconiellacamelliae* is synonymized with *Sinodisculacamellicola* while *Melanconiellaloropetali* is reclassified as *Sinodisculaloropetali*. The third species, *Melanconiellasyzygii*, forms a lineage with *Septomelanconiella*. However, the phylogenetic position of this isolate is not yet well-supported in the present analysis. Additional data are required to provide a more stable phylogenetic position, possibly leading to its reclassification under *Septomelanconiella*. The morphology of *M.syzygii* is similar to *Septomelanconiella* as they both possess septate conidia.

#### ﻿ Pseudomelanconiella

Taxon classificationFungiDiaporthalesMelanconiellaceae

Apurillo, Phukhams., E.B.G. Jones & K.D. Hyde gen. nov.

6DFC7A53-5C38-55A1-BC3A-05A554A774CD

Index Fungorum: IF902576

Facesoffungi Number: FoF16488

##### Etymology.

From the Greek “pseudo” meaning false, due to the close morphologic similarity of isolates with *Melanconiella* species.

##### Type species.

*Pseudomelanconiellamangrovei*.

##### Description.

**Sexual morph**: Undetermined. **Asexual morph: *Conidiomata*** globose to subglobose, immersed to erumpent, develop under a clypeus with variable stromata, confluent, mostly with a long, conical, central ostiole, black. ***Ostiole*** present. ***Peridium*** light brown to brown, composed of textura angularis cells. ***Conidiophores*** hyaline, septate. ***Conidiogenous cells*** hyaline, phialidic. ***Conidia*** oblong to ellipsoid, light-brown, unicellular, aseptate, verrucose, without gelatinous sheath.

##### Notes.

Combined phylogenetic analysis of ITS, LSU, *rpb2* and *tef1-α* sequences reveal that *Pseudomelanconiella* forms a distinct, well-supported clade separate from other genera in Melanconiellaceae. The sister taxon is *Septomelanconiella*, however, this monotypic genus is distinguished by its septate, laminate conidia, which is not observed in *Pseudomelanconiella* (Phookamsak et al. 2019). Both *Pseudomelanconiella* and *Septomelanconiella* are coelomycetous and have no reported sexual morphs (Phookamsak et al. 2019). Pairwise differences between the *Septomelanconiella* and *Pseudomelanconiella* in ITS (13.2%), LSU (1.6%), *rpb2* (16.1%) are all higher than the lowest base difference observed between genera in Melanconiellaceae. *Septomelanconiella* was isolated as a saprobe from *Syzygiumsamarangense* in a terrestrial environment while *Pseudomelanconiella* was isolated from mangroves in a brackish water environment (Phookamsak et al. 2019). These support the establishment of a new genus with *Pseudomelanconiellamangrovei* as the type species.

#### ﻿ Pseudomelanconiella mangrovei

Taxon classificationFungiDiaporthalesMelanconiellaceae

Apurillo, Phukhams., E.B.G. Jones & K.D. Hyde sp. nov.

4A0CA589-AE6E-5F30-A544-06B09DD6E7A6

Index Fungorum: IF902577

Facesoffungi Number: FoF16489

Fig. 2

##### Etymology.

Based on its mangrove host.

##### Holotype.

MFLU 24-0189.

##### Description.

***Saprobic*** on decomposing branch of ***Avicenniamarina*** submerged in brackish water. **Sexual morph**: Undetermined. **Asexual morph: *Conidiomata*** 100–590 µm × 200–815 µm (x̄ = 386.3 × 566.2 µm, n = 10), globose to subglobose, immersed to erumpent, black, confluent, mostly with a central stromatic column. ***Peridium*** light-brown to brown, made of cells of textura angularis. ***Conidiophores*** 8–20 × 1–2 µm (x̄ = 14.6 × 1.8 µm, n = 30), mostly straight, hyaline, septate, smooth unbranched. ***Conidiogenous cells*** 2–7 × 1–2 µm (x̄ = 4.2 × 1.8 µm, n = 30), monophialidic, determinate, discrete, cylindrical to subcylindrical, smooth-walled, hyaline, arising from inner layers of conidioma. ***Conidia*** 11–14 × 3–4 µm (x̄ = 11.4 × 3.3 µm, n = 50), oblong to ellipsoid, hyaline to light-brown, unicellular, aseptate, verrucose, without gelatinous sheath.

**Figure 2.**
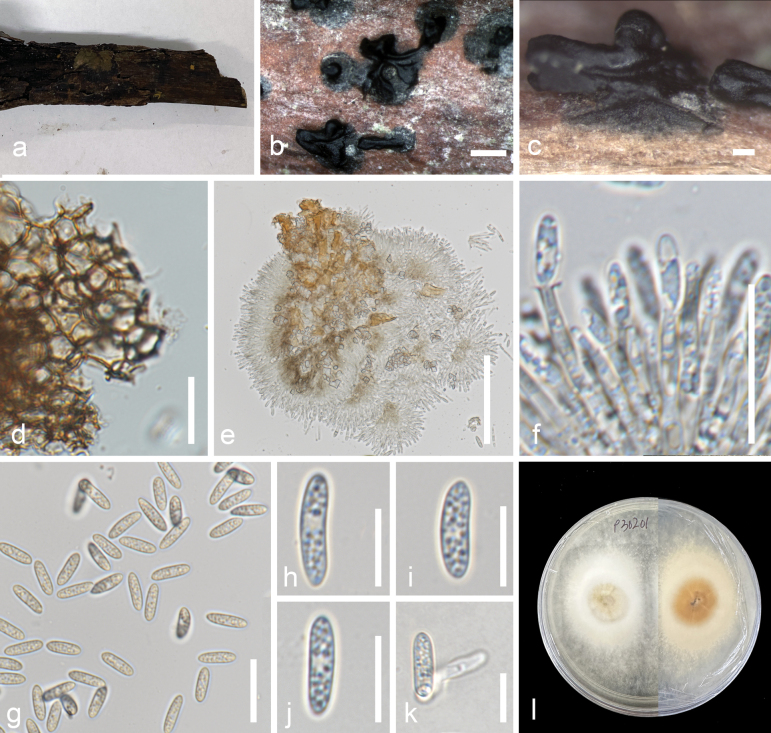
*Pseudomelanconiellamangrovei* (MFLU 24-0189, holotype). **a** host **b, c** conidiomata on host **d** section of peridium **e** squash mount showing conidiophores and conidia **f** conidiophores, conidiogenous cells with attached conidia **g–j** conidia **k** germinated conidium **l** culture on MEA. Scale bars: 500 µm (**b**); 100 µm (**c**); 50 µm (**e, f**); 20 µm (**d,g**); 10 µm (**h–k**).

##### Known distribution.

Thailand.

##### Culture characteristics.

Conidia germinate in malt extract agar (MEA) within 24 hours, with germ tubes arising from one end of the conidia. Colonies on MEA grow up to 14 cm after 7 days of incubation at room temperature, circular, mostly flat or effuse with a raised ring near the center, undulate, white, translucent; reverse does not exhibit pigments.

##### Material examined.

Thailand • Prachuap Khiri Khan Province, Pranburi District, 12°23'9"N, 99°56'51"E, on decomposing branch of *Avicenniamarina* L. (Acanthaceae) submerged in brackish water, 4 February 2023, Carlo Chris S. Apurillo, P30201 (MFLU 24-0189, ***holotype***); • ibid., P30301 (MFLU 24-0190); ex-type living culture MFLUCC 24-0512 = MFLUCC 24-0513 .

##### GenBank numbers.

MFLU 24-0189 = ITS: PP989291, LSU: PP989287, *rpb2*: PP993004, *tef*1-α: PP993001; MFLU 24-0190 = ITS: PP989292, LSU: PP989288, *tef*1-α: PP993002.

##### Notes.

Based on combined analysis of ITS, LSU, *rpb2*, and *tef1-α* sequences, *Pseudomelanconiellamangrovei* formed a distinct clade within Melanconiellaceae, with *Septomelanconiellathailandica* as the closest taxon. *Pseudomelanconiellamangrovei* differs from *Septomelanconiellathailandica* based on the appearance of the conidia. The distinguishing characteristic of *S.thailandica* is its septate conidia (Phookamsak et al. 2019). In contrast, *Pseudomelanconiellamangrovei* conidia are aseptate. While the morphology of *Pseudomelanconiellamangrovei* is similar to *Melanconiella* species, particularly the appearance of conidiomata, conidiogenous cells and the shape of the conidia, its phylogenetic position is distinct from other *Melanconiella* species (Voglmayr et al. 2012). Thus, this isolate is classified under a novel genus, *Pseudomelanconiella*.

### ﻿Sinodiscula M.J. Guo & C.L. Hou

#### ﻿ Sinodiscula loropetali

Taxon classificationFungiDiaporthalesMelanconiellaceae

(T.C. Mu & Jun Z. Qiu) Apurillo, Phukhams., K.D. Hyde & E.B.G. Jones comb. nov.

CD751322-6108-52FC-A758-F8C6BF1BD7AC

855635

Facesoffungi Number: FoF16612

Melanconiella loropetali T.C. Mu & Jun Z. Qiu, Front. Microbiol. 14(1229705):3 (2023). Basionym. MycoBank No: 848666.

##### Description.

**Sexual morph**: Undetermined. **Asexual morph**: Descriptions and illustrations refer to Mu et al. (2023).

##### Notes.

*Melanconiellaloropetali* was introduced in *Melanconiella* by Mu et al. (2023), isolated from diseased *Loropetalumsinense* in China. *Melanconiellaloropetali*, *Melanconiellacamelliae* and *Melanconiellasyzygii*, formed a basal clade distinct from other *Melanconiella* species in Mu et al. (2023). However, only *Melanconiella* species were used in the analysis, leading to their classification as *Melanconiella*. We included other genera in Melanconiellaceae in our analysis and *M.camelliae*, *M.loropetali* and *M.syzygii* are phylogenetically distant from *Melanconiella*. The phylogenetic tree of the combined ITS, LSU, *rpb2* and *tef1-α* sequences showed that *Melanconiellaloropetali* formed a sister clade to *Sinodiscula* species. Thus, we reclassified *M.loropetali* as *Sinodisculaloropetali*. *Melanconiellaloropetali* is similar to *Sinodiscula* species. It fits the generic description of *Sinodiscula*, and like other *Sinodiscula* species, *Melanconiellaloropetali* was isolated as a plant pathogen (Mu et al. 2023; Guo et al. 2024). Given the similarities in morphology and ecology of *M.loropetali* and *Sinodiscula* species, and the well-supported phylogenetic position of *Melanconiellaloropetali*, we reclassify it as *Sinodisculaloropetali*.

#### ﻿ Sinodiscula camellicola

Taxon classificationFungiDiaporthalesMelanconiellaceae

S.Y. Zhao, M.J. Guo & C.L. Hou, Journal of Fungi 10 (2, no. 141): 9 (2024)

3AC7349F-3422-5D5F-9A29-62DA5C72290E

Index Fungorum: IF851775

851775

≡ Melanconiellacamelliae T.C. Mu & Jun Z. Qiu, Front. Microbiol. 14(1229705):6 (2023). MycoBank No: 848667.

##### Description.

Descriptions and illustrations refer to Mu et al. (2023) and Guo et al. (2024).

##### Notes.

In the present analysis, *Melanconiellacamelliae* formed a well-supported clade with *Sinodisculacamellicola*. Detailed morphological comparison reveals that these two species are similar with no notable differences. Both species were isolated as pathogens from *Camelliasinensis*, from Fujian Province (China) for *Melanconiellacamelliae* and from Anhui Province (China) for *Sinodisculacamellicola* (Guo et al. 2024). Moreover, the ITS sequences of *Melanconiellacamelliae* and *Sinodisculacamellicola* differ by only 1%. Due to these similarities in morphology, ecology and molecular data of the two species, we propose to synonymize *M.camelliae* and *S.camellicola* under *Sinodiscula* (Melanconiellaceae).

### ﻿Xylariales Nannf.

**Diatrypaceae Nitschke**

**Peroneutypa Berl.**

Phylogenetic analysis of *Peroneutypa* was based on the combined ITS and *tub2* sequence data. The combined multi-locus analysis consisted of 927 characters, including gaps with the following lengths: ITS (1-531) and *tub2* (532-927). ML, MP, and BI analyses were done using single and concatenated sequences.

**Figure 3.**
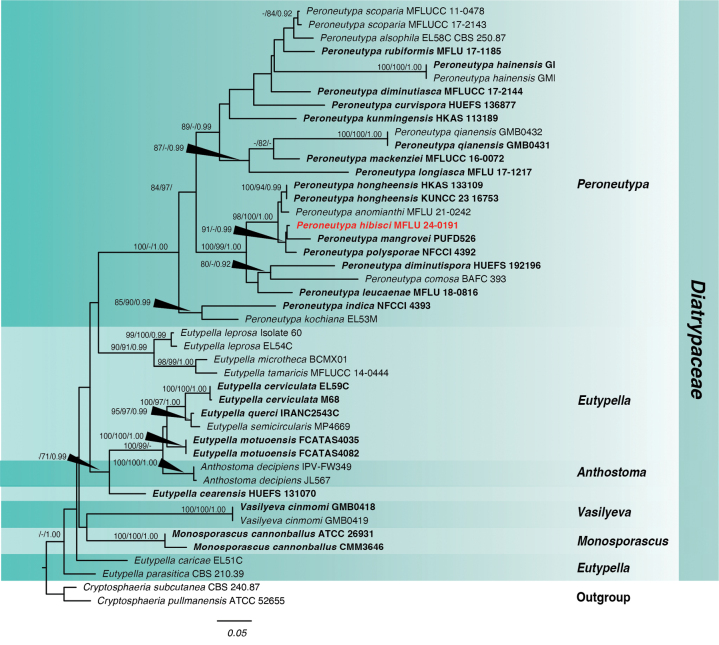
Phylogram of the combined ITS and *tub2* analysis of *Peroneutypa* and related genera in Diatrypaceae. Values above the branches indicate bootstrap support values from maximum likelihood (ML) and maximum parsimony (MP) equal to or above 0.70 and Bayesian posterior probability (PP) equal to or above 0.90. The novel species is indicated in red bold. Sequences from type species are indicated in bold. The tree is rooted with *Cryptoshaeriasubcutanea* CBS 240.87 *and Cryptosphaeria pullmanensis* ATCC 52655.

For ML, there were 540 distinct alignment patterns with 31.95% gaps and undetermined characters in the concatenated sequences. The maximum likelihood tree (Fig. 3) has a final ML optimization likelihood of -8,464.76, tree length of 3.225995 and alpha of 0.362736. The base frequencies are: A = 0.222507, C = 0.268440, G = 0.239063 and T = 0.269990. The rates of substitution are as follows: AC = 0.825859, AG = 2.247793, AT = 1.238868, CG = 0.697931, CT = 3.237131 and GT = 1.00000.

The MP analysis consisted of 927 total characters, 452 of which were constant, 396 parsimony-informative and 79 parsimony-uninformative. The most parsimonious tree had a similar topology as the best-scoring ML tree (Fig. 3).

For the Bayesian analysis, the model used for ITS was K2P+G and Hasegawa-Kishino-Yano with gamma distribution (HKY+G) for *tub2*. After the total MCMC generations, the average standard deviation of split frequencies is 0.002628. Analysis in Tracer showed that the two independent runs have converged based on the trace plots with the ESS values of all factors for the combined runs ranging from 4,877 to 14,599. The topology of the Bayesian tree is similar to the best-scoring tree in ML shown in Fig. 3, especially with respect to the position of *Peroneutypahibisci*.

The ML, MP, BI analyses showed that *Peroneutypahibisci* formed a distinct lineage with *Peroneutypamangrovei*, the latter showing a longer branch length (Fig. 3). Although the two species were isolated from mangroves, they differ in morphology as discussed in the taxonomy notes. Furthermore, the base pair difference in ITS and *tub2* are greater than 1.5%.

#### ﻿ Peroneutypa hibisci

Taxon classificationFungiXylarialesDiatrypaceae

Apurillo, Phukhams., E.B.G. Jones & K.D. Hyde sp. nov.

1F0ABEA7-52D2-5523-93D5-45B48F1E169B

Index Fungorum: IF902578

Facesoffungi Number: FoF16490

Fig. 4

##### Etymology.

Based on the host, *Hibiscus tiliaceus*.

##### Holotype.

MFLU 24-0191.

##### Description.

Saprobic on decomposing branches of ***Hibiscustiliaceus*** submerged in brackish water. **Sexual morph: *Stromata*** 1.0–1.5 mm (x̄ = 1.2, n = 5), poorly developed, non-sulcate, solitary to gregarious, immersed to erumpent, dark brown to black. ***Perithecia*** 200–300 µm (x̄ = 238, n = 5) in diameter, immersed to erumpent, globose, brown to black, ostiolate. ***Peridium*** 20–45 µm (x̄ = 32, n = 5) wide, composed of two layers, outer layer dark brown to black comprising of textura angularis cells, inner layer of textura angularis cells, brown to dark-brown. ***Hamathecium*** 90–125 µm × 3–4 µm (x̄ = 104 × 3.4 µm, n = 5) wide, hyaline, aseptate, unbranched. ***Asci*** 57–68 µm × 6–8 µm (x̄ = 59.4 × 7.6 µm, n = 5), 8-spored, clavate, unitunicate, short stipitate, with inamyloid apical rings. ***Ascospores*** 8–12 µm × 3–4 µm (x̄ = 10.7 × 3.6 µm, n = 45), hyaline, ellipsoid, with constricted median septum when mature, 2–4 guttules. **Asexual morph**: Undetermined.

##### Known distribution.

Thailand.

##### Culture characteristics.

Ascospores germinated in MEA within 24 hours, the germ tubes arising from both ends of the ascospore. Colonies on MEA grow up to 10 cm after 7 days of incubation at room temperature, white, filamentous, with aerial mycelium, reverse with gray pigment toward the center.

**Figure 4.**
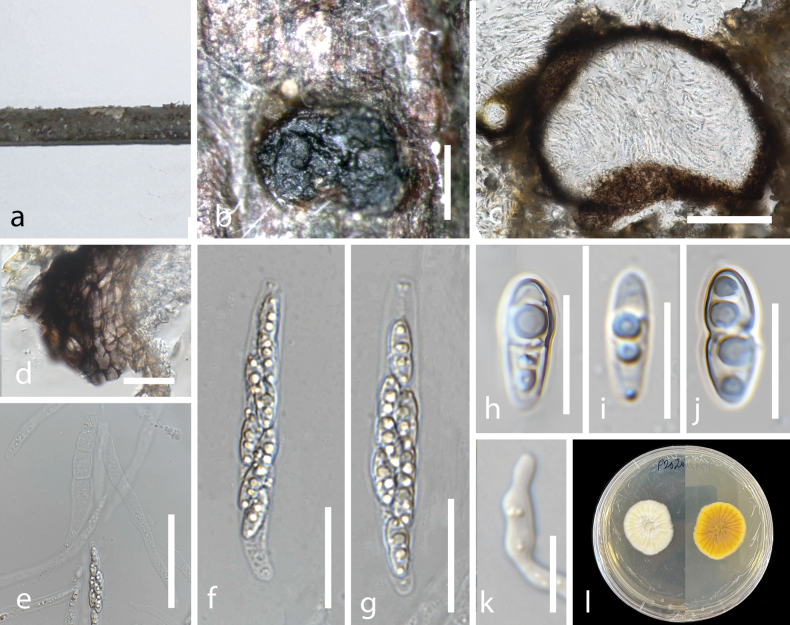
*Peroneutypahibisci* (MFLU 24-0191, holotype). **a** host **b** ascoma on host **c** section of ascoma **d** peridium **e** ascus with hamathecium **f, g** asci **h–j** ascospores **k** germinated ascospore **l** culture on MEA. Scale bars: 200 µm (**b**); 100 µm (**c**); 50 µm (**d**); 20 µm (**e, f**); 10 µm (**g–j**).

##### Material examined.

Thailand • Prachuap Khiri Khan: Pranburi District, 12°23'8.74"N, 99°56'51.47"E, on decomposing branches of *Hibiscustiliaceus* submerged in brackish water. 4 February 2023, Carlo Chris S. Apurillo, P20201 (MFLU 24-0191, ***holotype***), ex-type living culture, MFLUCC 24-0514.

##### GenBank Numbers.

ITS: PP989294, *tub2* = PP993003.

##### Notes.

*Peroneutypahibisci* formed a lineage with *Peroneutypamangrovei*, the latter showing a longer branch length than *Peroneutypahibisci*. Although this clade has low support, this was consistently observed in ML, MP and BI trees. Comparison of base pair differences between these two closely related species revealed a difference of 23 out of 459 bases (5.0%) in ITS and 7 out of 349 bases (2.0%) in *tub2* sequences. Morphologically, they differ significantly: *P.hibisci* has larger, ellipsoid, guttulate ascospores (8–12 × 3–4 µm), while *P.mangrovei* has smaller, cylindrical to clavate ascospores (3–5 × 1–1.5 µm) without guttules. The asci of *P.hibisci* (57–68 × 6–8 µm) are also much larger than those of *P.mangrovei* (14–20 × 3–4 µm) (Phookamsak et al. 2019). Compared to another related species, *P.polysporae*, *P.hibisci* differs by producing median-septate, ellipsoid ascospores, whereas *P.polysporae* has smaller, unicellular, allantoid spores. Additionally, *P.polysporae* has larger asci (110–155 × 5–7.5 µm) than *P.hibisci* (Dayarathne et al. 2020). Morphological and sequence data support the introduction of *Peroneutypahibisci* as a novel species based on the guidelines of Jeewon and Hyde (2016) and Maharachchikumbura et al. (2021).

### ﻿Dothideomycetes O.E. Erikss. & Winka

**Pleosporales Luttr. Ex M.E. Barr**

**Macrodiplodiopsidaceae Voglmayr, Jaklitsch & Crous**,

**Pseudochaetosphaeronema Punith.**

**Figure 5.**
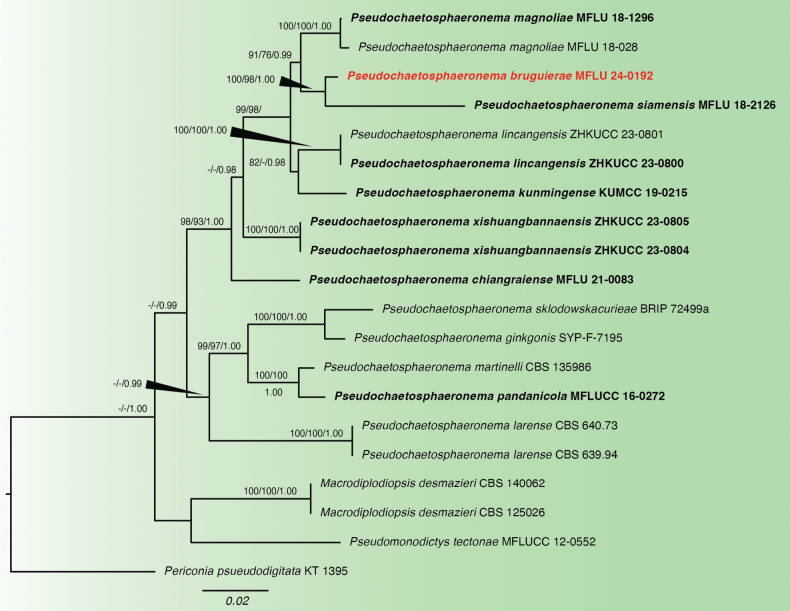
Phylogram of the combined ITS, LSU, SSU and *tef1-α* analysis of *Pseudochaetosphaeronema* and related genera. Values above the branches indicate bootstrap support values from maximum likelihood (ML) and maximum parsimony (MP) equal to or above 0.70 and Bayesian posterior probability (PP) equal to or above 0.90. The novel species is indicated in bold red. Sequences from type species are indicated in bold. The tree is rooted with *Periconiapseudodigitata*.

The ML, MP, and BI phylogenetic analyses of *Pseudochaetosphaeronema* utilized a combined multi-locus phylogeny including ITS, LSU, SSU, and *tef1-α* sequences. The alignment had a total of 3,313 characters, including gaps, with the lengths of the regions as follows: ITS (1-523), LSU (524-1,384), SSU (1,385-2,414), and *tef*1-α (2,415-3,313). For ML, the alignment has 607 distinct patterns with 20.91% gaps and completely undetermined characters. The best-scoring tree (Fig. 5) had a final optimization likelihood of -10,102.09 with tree length of 0.507278 and alpha = 0.103258. The base frequencies are as follows: A = 0.237153, C = 0.250803, G = 0.270592, T = 0.241452 and the following substitution rates: AC = 1.724291, AG = 4.038401, AT = 2.171255, CG = 1.954009, CT = 11.161818, GT = 1.00000.

For MP, out of the 3,313 characters in the dataset, 2,715 were constant, 368 were parsimony-informative while 230 were parsimony-uninformative. The topology of the ML tree from the combined analysis was similar to that of the best-scoring tree.

In the Bayesian analysis, the models used for the different loci were as follows: K2P+G for ITS and SSU, K2P+G+I for LSU and GTR+G+I for *tef1-α*. The average standard deviation of split frequencies after the total MCMC runs is 0.001383. Evaluation of the trace files in Tracer showed ESS values for all factors ranging 4,963 to 13,845. The plots showed that the two independent runs have converged. The topology of the BI tree was similar to the best-scoring ML tree, especially the position of *Pseudochaetosphaeronemabruguierae*.

Based on the ML, MP, and BI analyses, *Pseudochaetosphaeronemabruguierae* is found in a distinct, well-supported clade with *Pseudochaetosphaeronemasiamensis* as the sister taxon (Fig. 5). Notably, *P.siamensis* has a significantly longer branch length compared to *P.bruguierae*. Base differences in their sequences are also higher than 1.5%. In addition, *P.bruguierae* and *P.siamensis* differ in morphology of their conidiogenous cells and conidia, as discussed in the taxonomy notes.

#### ﻿ Pseudochaetosphaeronema bruguierae

Taxon classificationFungiPleosporalesMelanconiellaceae

Apurillo, Phukhams., E.B.G Jones & K.D. Hyde sp. nov.

C6A409AC-1BC8-5166-B04F-8C9ED65DAEF7

Index Fungorum: IF902579

Facesoffungi Number: FoF16491

Fig. 6

##### Etymology.

Based on the host *Bruguiera cylindrica*.

##### Holotype.

MFLU 24-0192.

##### Description.

***Saprobic*** on aerial dead branch of ***Bruguieracylindrica***. **Sexual morph**: Undetermined. **Asexual morph**: Coelomycetous. ***Conidiomata*** 230–400 × 300–370 µm diameter (x̄ = 344.5 × 339.0 µm, n = 10), dark brown to black, immersed to erumpent, solitary to gregarious, globose to subglobose, without ostiole. ***Conidiomata wall*** 22–66 µm, composed of dark brown, thick-walled cells. ***Conidiophores*** reduced to conidiogenous cells. ***Conidiogenous cells*** 7–10 µm × 1–2.7 µm (x̄ = 10.2 × 1.7 µm, n = 20), hyaline, cylindrical or ampulliform, monophialidic, smooth. ***Conidia*** 5–10 × 2–3 µm (x̄ = 6.5 × 2.2 µm, n = 30), hyaline to pale brown, fusiform, 2–3 septate, with constriction in the septa, with 26 guttules.

**Figure 6.**
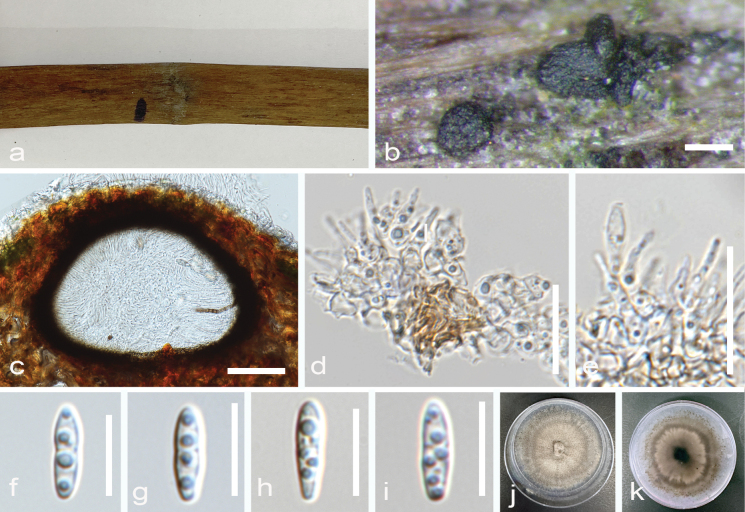
*Pseudochaetosphaeronemabruguierae* (MFLU 24-0192, *holotype*). **a** host **b** conidiomata on host **c** section of conidioma **d, e** conidiogenous cells **f–i** conidia **j** culture on MEA (obverse) **k** culture on MEA (reverse). Scale bars: 500 µm (**b**); 100 µm (**c**); 50 µm (**d, e**); 20 µm (**f**); 10 µm (**g–i**).

##### Known distribution.

Thailand.

##### Culture characteristics.

Conidia germinate in MEA within 24 hours, with germ tubes arising from one end of the conidia. Colonies on MEA grow up to 30 cm after 3 weeks of incubation at room temperature, circular, white, with light green pigment, flat or effuse, entire edge, reverse exhibits a dark green to pale brown pigmentation.

##### Material examined.

Thailand • Prachuap Khiri Khan Province: Pranburi District, 12°24'48"N, 99°56'51"E, on aerial dead branch of *Bruguieracylindrica* (Rhizophoraceae), 25 October 2022, Carlo Chris S. Apurillo, P11601 (MFLU 24-0192, ***holotype***), ex-type living culture MFLUCC 24-0515.

##### Genbank Numbers.

ITS: PP989295, LSU: PP989290, SSU: PP989296, *tef*1-α: PQ273803.

##### Notes.

Based on a combined phylogenetic analysis of ITS, LSU, SSU, and *tef1-α* sequence data, *Pseudochaetosphaeronemabruguierae* formed a clade with *Pseudochaetosphaeronemasiamensis*, with the latter showing longer branch length. Base pair comparison of ITS and *tef*1-α sequences revealed a 1.8% and 15% difference, respectively. The most significant difference between the two closely related species is their conidial morphology. *Pseudochaetosphaeronemabruguierae* has larger conidia (6.5 × 2.2 µm) which are fusiform, septate, with multiple guttules. In contrast, *Pseudochaetosphaeronemasiamensis* has smaller conidia (3 × 2 µm) which are subglobose to oval without septation (Jayasiri et al. 2019). Furthermore, the conidiogenous cells of *Pseudochaetosphaeronemasiamensis* are cylindrical with collarettes at the tips, while some appear flask-shaped with no collarettes for *Pseudochaetosphaeronemabruguierae* (Jayasiri et al. 2019). The conidiomata of *Pseudochaetosphaeronemabruguierae* is also larger than that of *Pseudochaetosphaeronemasiamensis*. *Pseudochaetosphaeronemabruguierae* is similar to *Pseudochaetosphaeronemamagnoliae* but they are phylogenetically distinct, with *P.magnoliae* forming a sister clade to *P.bruguierae* and *P.siamensis* (De Silva et al. 2022). Furthermore, *P.bruguierae* and *P.magnoliae* have a higher pairwise difference in the ITS sequences (9.3%). Although the shape of conidiogenous cells and the conidia of *P.bruguierae* and *P.magnoliae* are similar, they differ in terms of size, with *P.bruguierae* having larger conidiogenous cells and smaller conidia. Additionally, the conidia of *P.bruguierae* are septate with pronounced multiple guttules, which are not observed in the conidia of *P.magnoliae*. These support the introduction of *Pseudochaetosphaeronemabruguierae* as a novel species in *Pseudochaetosphaeronema* following the guidelines of Jeewon and Hyde (2016) and Maharachchikumbura et al. (2021).

### ﻿Pleosporales Luttr. ex M.E. Barr

**Aigialaceae Suetrong, Sakay., E.B.G. Jones, Kohlm., Volkm.-Kohlm. & C.L. Schoch**

**Rimora Kohlm., Volkm.-Kohlm., Suetrong, Sakay. & E.B.G. Jones**

Multi-locus analysis of *Rimora* was done using LSU, SSU and *tef1-α*. The alignment of concatenated sequences had a total length of 2,797 characters, including gaps. The specific lengths of each locus are as follows: LSU (1-853), SSU (854-1,876), *tef1-α* (1,877-2,797). In ML, the alignment has 720 distinct alignment patterns with 13.99% gaps and completely undetermined characters. After the analysis, the best-scoring ML tree (Fig. 5) had a final optimization value of -10,511.38, alpha = 0.162554 and tree length of 0.579899. The base frequencies are: A = 0.246335, C = 0.243918, G = 0.280712, and T = 0.229035 with the following substitution rates: AC = 1.97470, AG = 3.553768, AT = 1.000390, CG = 1.272768, CT = 13.114709 and GT = 1.00000.

For the BI analysis, different models were applied for each locus as follows: GTR+G for LSU and *tef1-α* and K2P+G+I for SSU. After the total MCMC generations, the average standard deviation of split frequencies is 0.003756. Analysis of trace files in Tracer showed ESS values ranging from 8,004 to 15,002 for the combined runs with the two independent runs showing convergence based on the plots. The topology of the BI tree was similar to the best-scoring ML tree (Fig. 5).

**Figure 7.**
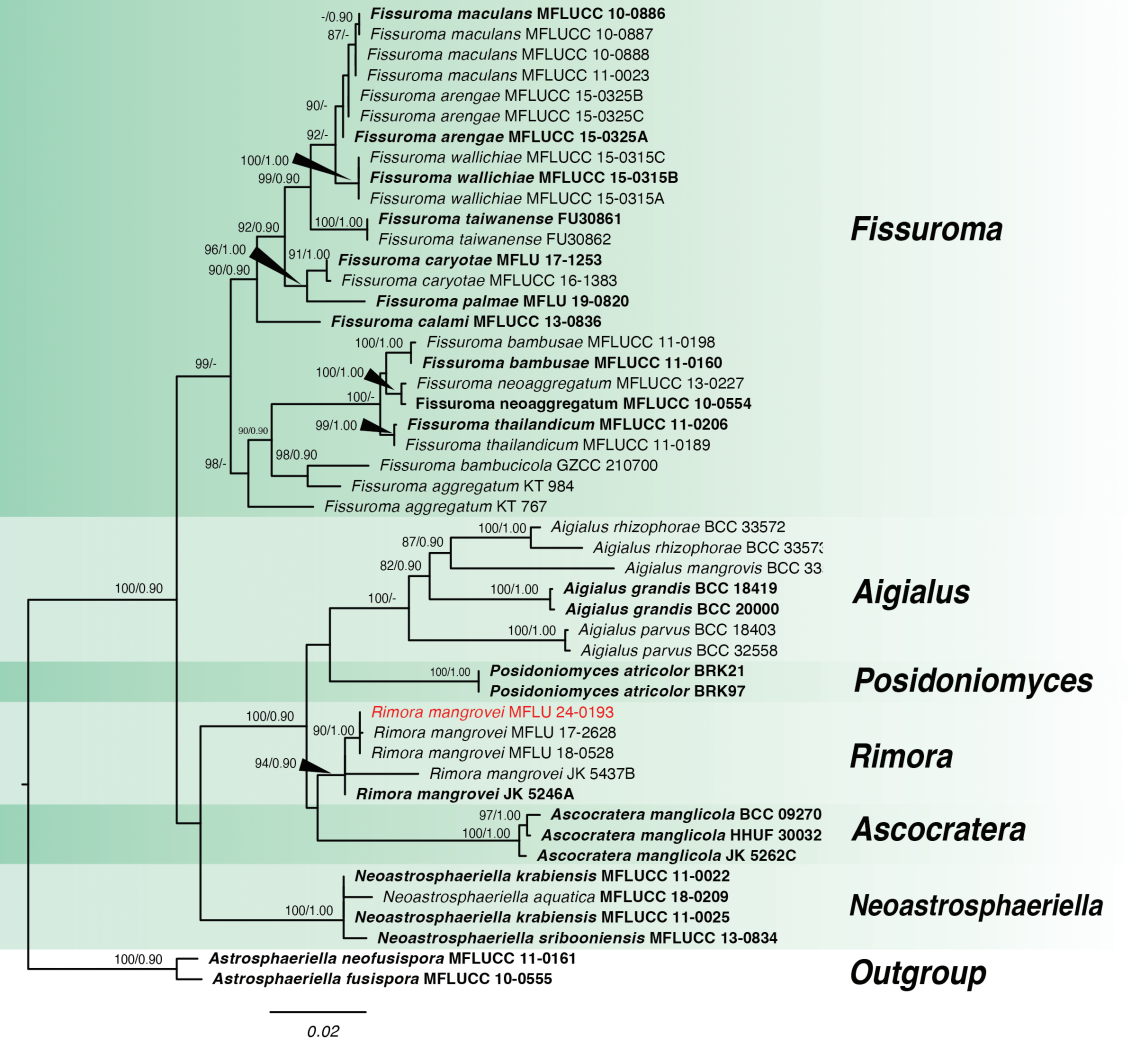
Phylogram of *Rimora* and closely-related genera based on a combined dataset of LSU, SSU and *tef1-α*. Values above the branches indicate bootstrap support from Maximum Likelihood (ML) and Bayesian posterior probability (PP). New record is indicated in red. Sequences from type species are indicated in bold. The tree is rooted with *Astroasphaeriella* species.

Based on the ML and BI multi-locus analyses, *Rimoramangrovei*MFLU 24-0193 is in a distinct clade with good support together with the holotype and other strains of *Rimoramangrovei* (Fig. 7). This strain, isolated from *Ceriopstagal*, represents a new host record for *Rimoramangrovei*.

#### ﻿ Rimora mangrovei

Taxon classificationFungiPleosporalesAigialaceae

(Kohlm. & Vittal) Kohlm., Volkm.-Kohlm., Suetrong, Sakay. & E.B.G. Jones

093FB376-784F-5E64-8C9F-8D07FA66D1AE

Index Fungorum: IF515959

515959

Facesoffungi Number: FoF08152

Fig. 8

##### Description.

***Saprobic*** on decomposing wood of ***Ceriops tagal*** submerged in brackish water. **Sexual morph**: ***Ascomata*** 336–634 µm × 200–483 (x̄ = 494 µm × 329 µm, n = 10) wide, globose to subglobose, black, carbonaceous, solitary to gregarious, immersed at first then later erumpent, with cleft-like ostiole, epapillate. ***Peridium*** 73–162 µm (x̄ = 112 µm, n = 10) thick, cells forming textura angularis. ***Pseudoparaphyses*** up to 2 µm, trabeculate (*sensu*Liew et al. 2000), branched, numerous. ***Asci*** 175–181 × 9–15 µm (x̄ = 178.8 × 12.4 µm, n = 10), 8-spored, bitunicate, cylindrical, short pedicellate, with an apical apparatus. ***Ascospores*** 40–47 µm × 7–11 µm (x̄ = 46.7 × 8.9 µm, n = 20), fusiform, hyaline, biseriate, 1-septate, constricted at the septum, with multiple prominent guttules. **Asexual morph**: Undetermined.

**Figure 8.**
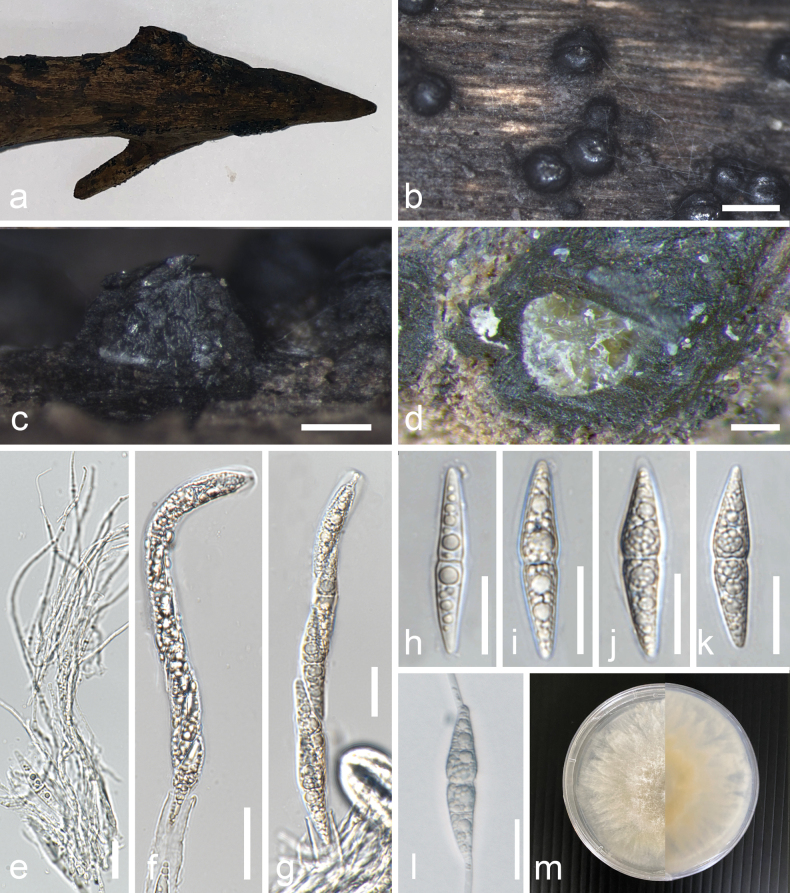
*Rimoramangrovei* (MFLU 24-0193). **a** host **b, c** ascomata on host **d** section of ascoma on host **e** paraphyses **f** immature ascus **g** mature ascus **h–k** ascospores **l** Germinated ascospore. m Colony on PDA. Scale bars: 500 µm (**b**); 200 µm (**c**); 250 µm (**d**); 20 µm (**e–l**).

##### Known distribution.

Thailand.

##### Culture characteristics.

Ascospores germinate in MEA within 24 hours, with germ tubes arising from one end of the ascospore. Colonies on MEA grow up to 6 cm after 14 days of incubation at room temperature, white, raised, fimbriated edge, reverse with light yellow pigment at the center which does not spread to the periphery.

##### Material examined.

Thailand • Prachuap Khiri Khan Province: Pranburi District, 12°24'48.672"N, 99°56'51.47"E, on decomposing wood of *Ceriopstagal* (Rhizophoraceae) submerged in brackish water, 25 October 2022, by Carlo Chris S. Apurillo, P10705 (MFLU 24-0193), ex-type living culture MFLUCC 24-0516.

##### GenBank Numbers.

ITS: PP989293, LSU: PP989289, SSU: PP989297.

##### Notes.

Based on a combined analysis of LSU, SSU and *tef1-α* sequences, *Rimoramangrovei*MFLU 24-0193 formed a well-supported clade with the holotype and other strains of *Rimoramangrovei* in Aigialaceae. This study identifies new characteristics not previously described for this species, including the presence of an apical apparatus in the asci and 1-septate ascospores with multiple prominent guttules. *Rimoramangrovei* has been reported from various mangrove hosts such as *Avicennia* species, *Bruguieragymnorrhiza*, *Ceriopsdecandra*, *Nypafruticans*, *Rhizophora* species, *and Sonneratia* species across countries in the Atlantic, Indian and Pacific regions (Devadatha et al. 2021). This is the first record of isolation of *Rimoramangrovei* from *Ceriopstagal* in Prachuap Khiri Khan, Thailand.

## ﻿Discussion

In this study, we introduce one novel genus, three novel species and one new record of fungi isolated from mangroves in Thailand. Two of these, *Pseudomelanconiellamangrovei* and *Pseudochaetosphaeronemabruguierae*, belong to genera not previously reported from mangroves. Based on morphological and molecular data, we establish *Pseudomelanconiella* as a novel genus in Melanconiellaceae (Diaporthales). This family is typified by *Melanconiella*, and also includes *Dicarpella*, *Greeneria*, *Melanconiella*, and *Microascospora* (Senanayake et al. 2017). Later additions included *Sheathospora* (Fan et al. 2018), *Septomelanconiella* (Phookamsak et al. 2019), *Paraphomopsis* (Udayanga et al. 2021), and most recently, *Sinodiscula* (Guo et al. 2024). Except for *Septomelanconiella*, all genera in this family contain species that cause plant diseases such as canker, dieback, leaf blight, and anthracnose (Du et al. 2017; Fan et al. 2018; Udayanga et al. 2021; Guo et al. 2024). However, *Pseudomelanconiella* is unique in being a saprobe in mangrove environments rather than a pathogen. While it groups with *Septomelanconiella*, it is distinct both morphologically and ecologically. Notably, it thrives in intertidal zones—an environment not previously reported for the family Melanconiellaceae.

*Melanconiellaloropetali* is reclassified as *Sinodisculaloropetali* and *Melanconiellacamelliae* was synonymized with *Sinodisculacamellicola*. *Melanconiella* species, typically found in Europe and North America on Betulaceae, have a narrow host and geographical range (Voglmayr et al. 2012). However, *M.syzygii* was reported from *Syzygium* sp. in Malaysia, the first time this genus was isolated outside Europe and North America from a different host (Crous et al. 2016). *Melanconiellaloropetali* and *M.camelliae* were also introduced to expand the host range of the genus (Mu et al. 2023). Our analysis confirms that *M.loropetali*, *M.camelliae* and *M.syzygii* do not belong to *Melanconiella*, thereby restricting the genus again to Betulaceae hosts (Voglmayr et al. 2012; Fan et al. 2018).

*Peroneutypahibisci*, a novel species, is also introduced in a genus with 43 known species (Index Fungorum 2024). Only two *Peroneutypa* species are from mangroves: *P.mangrovei* and *P.scoparia* while another two were reported from *Suaeda* plants in saline habitats: *P.indica* and *P.polysporae* (Jones et al. 2019; Devadatha et al. 2021). Our analysis groups *P.hibisci*, *P.mangrovei*, and *P.polysporae* together, suggesting that *Peroneutypa* species adapted to brackish or marine environments may have diverged separately from fungi in other ecological niches. For instance, these species may exhibit specialized traits such as salt tolerance or unique metabolic pathways for nutrient acquisition in saline conditions, distinguishing them from terrestrial *Peroneutypa* species that thrive in freshwater or soil habitats.

*Pseudochaetosphaeronemabruguierae* is also a novel species, the first in the genus to be reported from mangroves. *Pseudochaetosphaeronema* was established in 1979 to accommodate *P.larense* (≡ *Chaetosphaeronemalarense*), a pathogenic fungus isolated from human foot (Punithalingham 1979). This remained a monotypic genus until 2015 when Ahmed et al. (2015) introduced *Pseudochaetosphaeronemamartinelli*, isolated as a pathogen in immunosuppressed humans. Currently, there are 12 species recorded for this genus, many of which have been isolated as saprobes of terrestrial plants and soil (Phookamsak et al. 2019; Boonmee et al. 2021; Tan and Shivas 2022; Xu et al. 2024). Although *Pseudochaetosphaeronema* is known as a human pathogen due to the first two species introduced, the other members of this genus are saprobes. Notably, none have been isolated from mangroves. In this study, *Pseudochaetosphaeronemabruguierae* was isolated from the dead branch of a mangrove that was not submerged in brackish water, thus, it is not considered a marine fungus. Currently, there is no evidence that the isolate can survive in a saline environment. Initially classified as *incertae sedis* in Pleosporales (Dothideomycetes), *Pseudochaetosphaeronema* was later accepted under Macrodiplodiopsidaceae (Pleosporales) by Wijayawardene et al. (2022).

*Rimoramangrovei* is introduced with *Ceriopstagal* as a new host record. *Rimora* is a monotypic genus typified by *Rimoramangrovei* (Suetrong et al. 2009). *Rimora* is one of three genera in Aigialaceae alongside *Aigialus* and *Ascocratera*, whose type species were first isolated from mangroves (Kohlmeyer and Schatz 1985; Kohlmeyer 1986). Another species in Aigialaceae, *Posidoniomycesatricolor*, the only species in the genus, was isolated as an endophyte of seagrass (Vohník et al. 2019). These examples highlight that many members of Aigialaceae can survive in marine environment, establishing it as a well-known marine family. The addition of a new record from mangroves in this family further confirms the ecological niche of Aigialaceae.

In conclusion, this study underscores the vital importance of ongoing exploration in mangrove ecosystems to uncover the diverse fungal species and their significance (Hyde and Lee 1995; Rampadarath et al. 2018). Despite considerable research, numerous fungal species in mangroves remain undiscovered. The discovery of novel genus and species, such as *Pseudomelanconiella*, *Peroneutypa*, *Pseudochaetosphaeronema*, highlights the species richness of these environments. Further exploration is crucial not only for taxonomic understanding but also for discovering potential bioactive compounds with pharmaceutical and biotechnological applications. Indeed, many fungi from mangroves, including endophytes, have been reported to possess bioactive compounds with antibacterial, antimutagenic, and antioxidant properties among others (Dela Cruz et al. 2020; Cadamuro et al. 2021; Sopalun et al. 2021). This study emphasizes the necessity of sustained efforts in studying mangrove fungi, both as a means of understanding ecological interactions and for potential beneficial discoveries.

## Supplementary Material

XML Treatment for Pseudomelanconiella

XML Treatment for Pseudomelanconiella mangrovei

XML Treatment for Sinodiscula loropetali

XML Treatment for Sinodiscula camellicola

XML Treatment for Peroneutypa hibisci

XML Treatment for Pseudochaetosphaeronema bruguierae

XML Treatment for Rimora mangrovei

## ﻿Additional information

### Author ORCIDs

Carlo Chris S. Apurillo https://orcid.org/0000-0003-4348-0887

Chayanard Phukhamsakda https://orcid.org/0000-0002-1033-937X

Kevin D. Hyde https://orcid.org/0000-0002-2191-0762

Vinodhini Thiyagaraja https://orcid.org/0000-0002-8091-4579

E. B. Gareth Jones https://orcid.org/0000-0002-7286-5471

### Data availability

All of the data that support the findings of this study are available in the main text or Supplementary Information.

## Supplementary materials

Supplementary material 1 Additional information Data type xlsx Explanation note **table S1.** Genbank accession codes for the sequences of Melanconiellaceae species and outgroup used in this study. **table S2.** Genbank accession numbers of *Peroneutypa* and related genera used in this study. **table S3.** Genbank accession numbers of Pseudochaetosphaeronema and related genera used in this study. **table S4.** Genbank accession numbers of Aigialaceae strains used in this study. https://binary.pensoft.net/file/1301068This dataset is made available under the Open Database License (http://opendatacommons.org/licenses/odbl/1.0/). The Open Database License (ODbL) is a license agreement intended to allow users to freely share, modify, and use this Dataset while maintaining this same freedom for others, provided that the original source and author(s) are credited.Carlo Chris S. Apurillo, Chayanard Phukhamsakda, Kevin D. Hyde, Vinodhini Thiyagaraja, E. B. Gareth Jones
